# Report on Filling Teeth

**Published:** 1872-11

**Authors:** J. C. Ross


### Report on Filling Teeth.

BY J. C. ROSS, D. D. S.

The preservation of the natural teeth should be the first object of every dental practitioner, and as plugging teeth is the principal means employed to that end, and as to *fill teeth well* is the highest attainment of the profession, and at the same time one of the most difficult to perform, whatever means and appliances are invented or discovered which may tend to facilitate the dentist in his operations, by enabling him to perform them in less time and more perfectly, is that much of a contribution to science and benefit to his patients.

After the cavity in a tooth has been properly excavated, in order to perfect success in filling, it should be thoroughly dry, and kept dry during the process of filling.

For this purpose the rubber dam has been found to be the most efficient appliance known to the profession. By many it has come to be regarded as almost indispensable especially in contour fillings. In some instances, until we have more experience, it will be found difficult of application, especially on the molar teeth.

To Dr. S. C. Barnum, of New York, we are indebted for the rubber dam. In 1864 he made a free tender of his most valuable discovery tó the dental profession.

As a means of preventing moisture while operating, its utility and efficiency can scarcely be over-estimated.

A most valuable iustrument for preparing cavities for filling teeth, for cleansing the teeth, and especially for finishing fillings and other operations, is the "Dental Engine." We refer particularly to the one recently invented by Dr. J. B. Morrison, of St. Louis.

Though introduced to the profession only about a year ago, its utility and efficiency have been satisfactorily tested. It is a valuable acquistion to the dental office.

Johnson Brothers, of New York, are the sole manufacturers and vendors; and as gotten up by them, besides being a valuable instrument, it is a delicate and beautiful piece of, mechanism.

The "Pneumatic Burring Engine," invented by G. F. Green, of Kalamazoo, Michigan, was introduced to the profession some three years ago—an instrument for preparing cavities, finishing fillings, etc.; but the Morrison, as its cost is less, and being lighter, more delicate, and entirely noiseless, is likely to be preferred if not to supercede the pneumatic.

As to the material for filling teeth—though in the last few years a great many kinds have been introduced to the profession, some of which serve a valuable purpose in cases where the teeth are badly wasted from decay, and expense to the patient is an object; but whenever the condition of the tooth to be filled, and the circumstances of the patient will justify, gold is to be preferred—not only preferred, but to hold the pre-eminence.

